# Willingness to Use Home Collection Methods to Provide Specimens for SARS-CoV-2/COVID-19 Research: Survey Study

**DOI:** 10.2196/19471

**Published:** 2020-09-03

**Authors:** Eric William Hall, Nicole Luisi, Maria Zlotorzynska, Gretchen Wilde, Patrick Sullivan, Travis Sanchez, Heather Bradley, Aaron J Siegler

**Affiliations:** 1 Department of Epidemiology Rollins School of Public Health Emory University Atlanta, GA United States; 2 Department of Epidemiology & Biostatistics School of Public Health Georgia State University Atlanta, GA United States; 3 Department of Behavioral, Social and Health Education Sciences Rollins School of Public Health Emory University Atlanta, GA United States

**Keywords:** COVID-19, SARS-CoV-2, specimen collection, survey, research, public health, infectious disease, virus, test

## Abstract

**Background:**

Innovative laboratory testing approaches for SARS-CoV-2 infection and immune response are needed to conduct research to establish estimates of prevalence and incidence. Self-specimen collection methods have been successfully used in HIV and sexually transmitted infection research and can provide a feasible opportunity to scale up SARS-CoV-2 testing for research purposes.

**Objective:**

The aim of this study was to assess the willingness of adults to use different specimen collection modalities for themselves and children as part of a COVID-19 research study.

**Methods:**

Between March 27 and April 1, 2020, we recruited 1435 adults aged 18 years or older though social media advertisements. Participants completed a survey that included 5-point Likert scale items stating how willing they were to use the following specimen collection testing modalities as part of a research study: home collection of a saliva sample, home collection of a throat swab, home finger-prick blood collection, drive-through site throat swab, clinic throat swab, and clinic blood collection. Additionally, participants indicated how the availability of home-based collection methods would impact their willingness to participate compared to drive-through and clinic-based specimen collection. We used Kruskal-Wallis tests and Spearman rank correlations to assess if willingness to use each testing modality differed by demographic variables and characteristics of interest. We compared the overall willingness to use each testing modality and estimated effect sizes with Cohen *d*.

**Results:**

We analyzed responses from 1435 participants with a median age of 40.0 (SD=18.2) years and over half of which were female (761/1435, 53.0%). Most participants agreed or strongly agreed that they would be willing to use specimens self-collected at home to participate in research, including willingness to collect a saliva sample (1259/1435, 87.7%) or a throat swab (1191/1435, 83.1%). Willingness to collect a throat swab sample was lower in both a drive-through setting (64%) and clinic setting (53%). Overall, 69.0% (990/1435) of participants said they would be more likely to participate in a research study if they could provide a saliva sample or throat swab at home compared to going to a drive-through site; only 4.4% (63/1435) of participants said they would be less likely to participate using self-collected samples. For each specimen collection modality, willingness to collect specimens from children for research was lower than willingness to use on oneself, but the ranked order of modalities was similar.

**Conclusions:**

Most participants were willing to participate in a COVID-19 research study that involves laboratory testing; however, there was a strong preference for home specimen collection procedures over drive-through or clinic-based testing. To increase participation and minimize bias, epidemiologic research studies of SARS-CoV-2 infection and immune response should consider home specimen collection methods.

## Introduction

The first case of the novel coronavirus SARS-CoV-2 in the United States was identified on January 20, 2020 [1]. By April 8, the number of reported cases in the United States had surpassed 400,000 [2]. Over that same time period, roughly 2 million specimens had been tested for SARS-CoV-2 RNA in laboratories across the country [3]. While increased laboratory capacity and the opening of drive-through facilities has increased testing access and case identification, these strategies remain insufficient to support the population-based research required to characterize the epidemiologic nature of this outbreak because testing is focused in clinical settings and on people with symptoms of disease. To develop a better understanding of the exposure, disease, and recovery process associated with SARS-CoV-2 infection, infectious disease researchers have called for innovative testing approaches and a rapid scaleup in the number of persons tested [4].

Self-specimen collection for testing has been successfully used in HIV and sexually transmitted infection (STI) research for well over a decade [5-8]. A review of 25 HIV testing studies found that across multiple specimen methods (finger prick, oral swabs), self-collection results had the same diagnostic accuracy as clinician-collected specimens, with no differences in the proportions of invalid results [9]. Another review comparing participant self-collection versus clinician collection for gonorrhea and chlamydia also found high performance (>90% sensitivity and specificity) for self-collected specimens [10]. In one of our previous studies, 93% of participants were able to successfully complete multiple specimen collections, and 85% preferred self-collection of specimens at home to a standard office visit [11]. In the present analysis, we aimed to assess the willingness of adults to use different specimen collection methods on themselves or their children as part of a COVID-19 research study. We hypothesized that modalities for home specimen collection would be preferred over clinic-based specimen collection.

## Methods

### Recruitment

Participants were recruited through web-based social media advertisements on Facebook, Snapchat, and Twitter from March 27, 2020 to April 1, 2020. Internet users who clicked on the advertisements were taken to a consent module and short screener to determine eligibility. Eligible respondents were adults aged ≥18 years. On the last day of recruitment, we oversampled Hispanic and Black respondents with targeted ads to increase the racial and ethnic diversity of the sample. Eligible participants completed a web-based survey that collected data on their demographics, current knowledge of COVID-19, stigma related to COVID-19, and relevant symptoms over the last 24 hours. We used cookie-based duplicate protection, which restricts respondents from completing the survey more than once from the same browser on the same device. Participants were not compensated for their participation.

Next, the participants answered a series of 5-point Likert scale items (1=strongly disagree, 2=disagree, 3=undecided, 4=agree, 5=strongly agree) about their willingness to use different specimen collection modalities to test for SARS-CoV-2 infection as part of a research study. Participants who indicated having children aged <18 years in their household were also asked about their willingness to use the same modalities to collect specimens from their child as part of a research study. The modalities included home collection of a saliva sample, home throat swab collection, home finger prick blood collection, drive-through site throat swab collection, clinic throat swab collection, and clinic blood collection. The questions indicated that all specimens collected at home would be mailed to a central laboratory for testing. The definitions provided to participants for each testing modality are reported in Table 1.

Finally, the participants were asked how the availability of a home specimen collection method to test for COVID-19 that used either a saliva sample or throat swab would impact their willingness to participate in a research study compared to a drive-through sample collection site and a clinic sample site. For these questions, possible answers included “more likely to participate in a research study,” “about the same likelihood to participate in a research study,” and “less likely to participate in a research study.”

**Table 1:** Definitions of specimen collection testing modalities used in a web-based survey to assess willingness to participate in a COVID-19 research study in the United States in March 2020.

Testing modality	Survey definition
Home saliva sample	A home saliva sample would involve you spitting in a tube and sending it to a certified laboratory.
Home throat swab	A home throat swab would involve you using a throat swab and sending it to a certified laboratory.
Home blood collection	A home blood test would involve using an automated finger prick device, collecting a blood sample on a specimen card, and mailing in a prepaid mailer to a certified laboratory.
Drive-through site throat swab	A drive-through site for throat swab would involve your traveling to a drive-through facility in your car to have a health care worker collect the swab.
Clinic throat swab	A laboratory throat swab would involve your traveling to a laboratory facility in a clinic or private laboratory to have a health care worker collect the swab.
Clinic blood collection	A laboratory blood test would involve your traveling to a laboratory facility in a clinic or a private lab to have blood drawn, similar to a usual doctor's visit.

### Statistical Analysis

All analysis was performed using RStudio v1.1.453. To present a complete description of these data, we summarized the participants’ willingness to use each testing modality by calculating both the mean (SD) and median (IQR). A stigma index score was calculated by summing the number of stigma-related items the participant indicated as true (maximum=4). Similarly, we calculated a knowledge index score by tabulating the number of correct responses to the knowledge items (maximum=14). In the methodological literature, there is an ongoing debate about whether parametric or nonparametric statistical methods should be used for Likert-type data [12,13]; therefore, we explored the data with both methods. First, we used nonparametric Kruskal-Wallis tests to assess if willingness to use each testing modality differed by categorical demographic variables and nonparametric Spearman rank correlation coefficients to assess if willingness differed by ordinal characteristics (eg, income, education, likelihood of currently having COVID-19, stigma index score, knowledge index score, and number of symptoms in the past 24 hours). Second, we used parametric statistical methods to facilitate interpretation of the main findings, using Cohen *d* to estimate the effect size of the overall willingness to use each testing modality. Cohen *d* reports the estimated difference in mean values in terms of SD [14]. For example, a Cohen *d* of 1 indicates that the mean of one group is one standard deviation away from than the mean of the comparison group. All *P* values were adjusted for multiple tests using the Bonferroni-Holm method.

## Results

A total of 4593 respondents started the eligibility screener. Of these, 12 (0.3%) were removed for duplicate IP addresses, 1260 (27.4%) did not meet the eligibility criteria, and 1886 (41.1%) failed to complete the primary outcome survey questions, resulting in an analytic dataset of 1435 (31.2%) survey responses. The demographic characteristics are summarized in Table 2. Over half the participants (761/1435, 53.0%) were female, and the mean age was 40.0 years (SD 18.2). Many participants were non-Hispanic White (587/1435, 40.9%) or Hispanic (548/1435, 38.2%), and most had either completed a college degree (629/1435, 43.8%) or attended some college, associate degree, or technical school (382/1435, 26.6%). Over one-quarter of respondents (385/1435, 26.8%) reported children aged <18 years in their household and answered survey questions about their willingness to use different specimen collection modalities for SARS-CoV-2 testing to collect specimens from their children.

**Figure 1:**
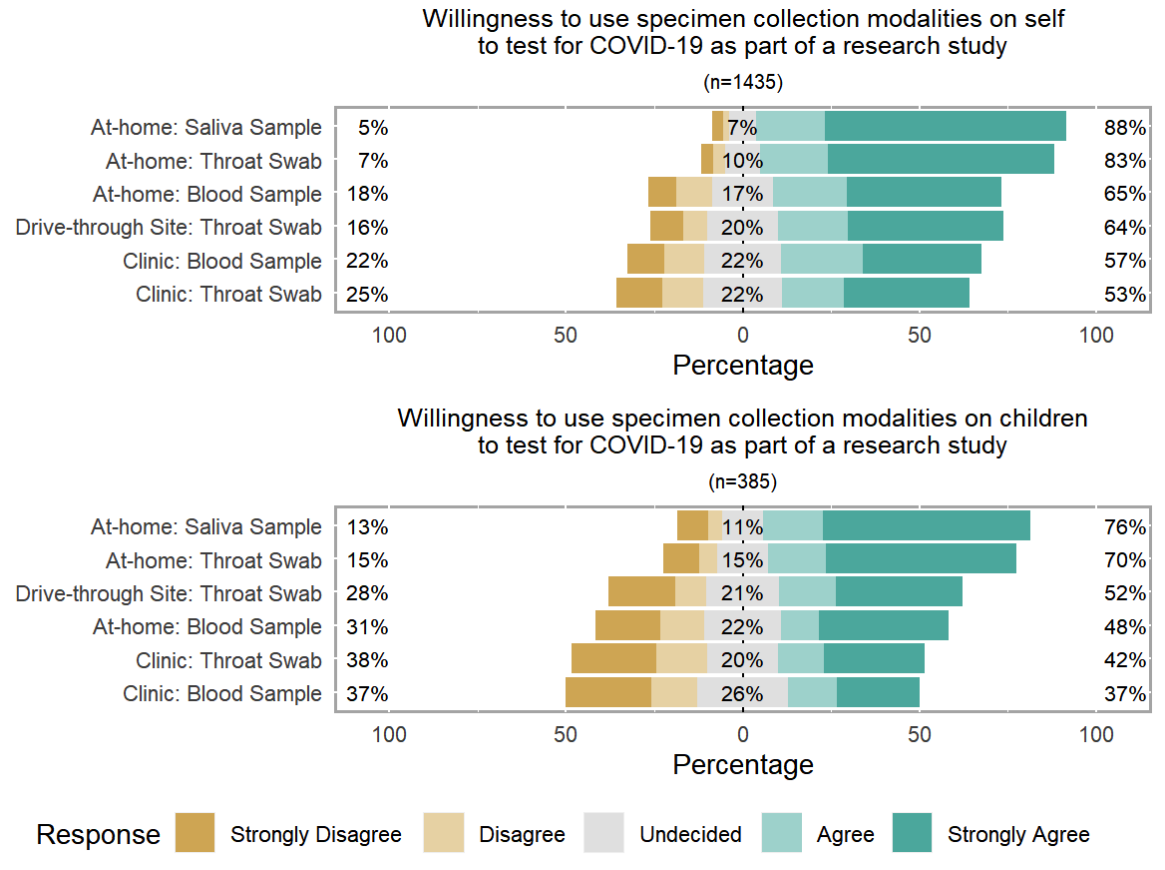
Stated willingness to use testing modalities as part of a COVID-19 research study by internet-using adults aged ≥18 years in the United States in March 2020 on themselves (top) and on their children (bottom). All specimen collection modalities involved testing the specimens in a central laboratory.

Figure 1 displays the participants’ stated willingness to use different specimen collection modalities for SARS-CoV-2 testing on themselves and their children. Overall, the large majority of participants agreed or strongly agreed that they would be willing to use a home specimen collection method to obtain a saliva sample (1259/1435, 87.7%) or a throat swab (1191/1435, 83.1%) from themselves as part of a research study. More than half the participants agreed or strongly agreed that they would be willing to acquire a home specimen collection finger prick blood sample (928/1435, 64.7%), visit a drive-through site to provide a throat swab (914/1435, 63.7%), or visit a clinic to provide a blood sample (812/1435, 56.6%) or a throat swab (762/1435, 53.1%). In a separate question about relative preference between multiple specimen collection modalities, 990/1435 participants (69.0%) said they would be more likely to participate in a research study if they could collect a saliva sample or throat swab at home compared to going to a drive-through site. Similarly, 1023/1435 participants (71.3%) stated that they would be more likely to participate in a research study with specimens to be collected at home compared to a study with specimens collected at a clinic. Of the 1435 participants, only 63 (4.4%) and 82 (5.7%) reported that using a home specimen collection process would make them less likely to participate in a research study compared to sample collection at a drive-through site or a clinic, respectively.

Relative to the participants’ willingness to participate in research themselves, their willingness to have their children participate in research was lower for each specimen collection modality (Figure 1). The proportion of participants willing to use each modality to collect specimens from their children ranged from 291/385 (75.6%) who were willing to perform home collection of a saliva sample to 124/334 (37.1%) who were willing to take their child to a clinic for a blood sample. However, the ranked orders of the participants’ willingness to use each testing modality on themselves and on their children were similar.

For most comparisons, the stated willingness to use each specimen collection modality did not differ by demographic group, stigma index score, or presence of current COVID-19 symptoms (Table 2, Table 3, and Table 4). A notable exception was that younger participants were slightly less willing to obtain a home-collected throat swab (adjusted *P*=.049) or visit a drive-through site to provide a throat swab (adjusted *P*=.047). While there was no difference in willingness to use home collection saliva samples or throat swabs, participants who thought it was somewhat likely, likely, or very likely that they currently had COVID-19 had moderately higher willingness to visit a drive-through site (adjusted *P*=.01) or a clinic (adjusted *P*=.003) to provide a throat swab.

**Table 2:** Demographic characteristics of the study participants (internet-using adults aged >18 years in the United States in March 2020) and their stated willingness to use home saliva sample and throat swab specimen collection testing modalities on themselves as part of a COVID-19 research study (N=1435). All survey questions were 5-point Likert scale items where 1=strongly disagree, 2=disagree, 3=undecided, 4=agree, 5=strongly agree. Kruskal-Wallis tests and Spearman rank correlation coefficients were used to assess response differences by characteristic.

Characteristic		n (%)		Home: saliva sample						Home: throat swab								
					Mean (SD)		Median (IQR)		*P* value^a^		Mean (SD)			Median (IQR)				*P* value
Overall		1435 (100.0)		4.5 (0.9)		5 (4-5)		N/A^b^		4.4 (1.9)			5 (4-5)				N/A	
**Gender**								>.99									>.99	
	Female		761 (53.0)		4.5 (0.9)		5 (4-5)				4.3 (1.0)			5 (4-5)				
	Male		536 (37.4)		4.5 (0.9)		5 (4-5)				4.4 (1.0)			5 (4-5)				
	Other		36 (2.5)		4.6 (1.0)		5 (5-5)				4.5 (1.0)			5 (4.75-5)				
**Age (years)**								.14									.049	
	18-29		560 (39.0)		4.4 (1.0)		5 (4-5)				4.2 (1.1)			5 (4-5)				
	30-49		391 (27.2)		4.5 (0.9)		5 (4-5)				4.4 (1.0)			5 (4-5)				
	50-64		289 (20.1)		4.6 (0.7)		5 (4-5)				4.5 (0.9)			5 (4-5)				
	≥65		194 (13.5)		4.5 (0.9)		5 (4-5)				4.4 (1.0)			5 (4-5)				
**Race/ethnicity**								.06									>.99	
	Hispanic		548 (38.2)		4.5 (0.9)		5 (4-5)				4.4 (1.0)			5 (4-5)				
	Asian/Pacific Islander		52 (3.6)		4.6 (0.7)		5 (4-5)				4.5 (0.8)			5 (4-5)				
	Non-Hispanic Black		158 (11.0)		4.3 (1.0)		5 (4-5)				4.2 (1.1)			5 (4-5)				
	Non-Hispanic White		587 (40.9)		4.6 (0.9)		5 (4-5)				4.4 (1.0)			5 (4-5)				
	Other		90 (6.2)		4.3 (1.1)		5 (4-5)				4.1 (1.3)			5 (4-5)				
**Education**								>.99									>.99	
	College, postgraduate, or professional school		629 (43.8)		4.5 (0.9)		5 (4-5)				4.4 (1.0)			5 (4-5)				
	Some college, associate's degree, or technical school		382 (26.6)		4.6 (0.8)		5 (4-5)				4.5 (0.9)			5 (4-5)				
	High school/GED^c^		175 (12.2)		4.4 (1.0)		5 (4-5)				4.3 (1.1)			5 (4-5)				
	Did not finish high school		27 (1.9)		4.6 (0.9)		5 (4-5)				4.4 (1.0)			5 (4-5)				
**Annual income (US $)**								.44									>.99	
	<24,000		294 (20.5)		4.5 (0.9)		5 (4-5)				4.3 (1.1)			5 (4-5)				
	24,000 to <50,000		276 (19.2)		4.5 (1.0)		5 (4-5)				4.4 (1.1)			5 (4-5)				
	50,000 to <75,000		203 (14.1)		4.6 (0.9)		5 (4-5)				4.5 (0.9)			5 (4-5)				
	≥75,000		268 (18.7)		4.7 (0.8)		5 (5-5)				4.5 (0.9)			5 (4-5)				
	Don't know		91 (6.3)		4.5 (0.8)		5 (4-5)				4.3 (0.9)			5 (4-5)				
**How likely do you think it is you have COVID-19 now?**								>.99									>.99	
	Very unlikely		356 (24.8)		4.4 (1.1)		5 (4-5)				4.3 (1.2)			5 (4-5)				
	Unlikely		661 (46.1)		4.5 (0.9)		5 (4-5)				4.3 (1.0)			5 (4-5)				
	Somewhat likely		324 (22.6)		4.5 (0.8)		5 (4-5)				4.5 (0.9)			5 (4-5)				
	Likely/very likely		81 (5.6)		4.5 (0.8)		5 (4-5)				4.5 (0.9)			5 (4-5)				
**Stigma index score**								.70									>.99	
	0		722 (50.3)		4.5 (0.9)		5 (4-5)				4.4 (1.0)			5 (4-5)				
	1-2		525 (36.6)		4.5 (1.0)		5 (4-5)				4.4 (1.0)			5 (4-5)				
	≥3		106 (7.4)		4.3 (1.1)		5 (4-5)				4.3 (1.2)			5 (4-5)				
**Knowledge index score**								.02									>.99	
	<12		337 (23.5)		4.4 (0.9)		5 (4-5)				4.4 (1.0)			5 (4-5)				
	12-13		655 (45.6)		4.5 (1.0)		5 (4-5)				4.4 (1.1)			5 (4-5)				
	14		342 (23.8)		4.5 (0.9)		5 (4-5)				4.4 (1.0)			5 (4-5)				
**Symptoms**								.71									>.99	
	1 or more symptoms		747 (52.1)		4.5 (0.9)		5 (4-5)				4.4 (1.0)			5 (4-5)				
	None		688 (47.9)		4.4 (1.0)		5 (4-5)				4.3 (1.1)			5 (4-5)				

^a^*P* values were adjusted for multiple comparisons using the Bonferroni-Holm method.

^b^N/A: not applicable.

^c^GED: General Education Development.

**Table 3:** Stated willingness of internet-using adults aged ≥18 years in the United States in March 2020 to use drive-through and clinic throat swab specimen collection testing modalities on themselves as part of a COVID-19 research study (N=1435). All survey questions were 5-point Likert scale items where 1=strongly disagree, 2=disagree, 3=undecided, 4=agree, 5=strongly agree. Kruskal-Wallis tests and Spearman rank correlation coefficients were used to assess response differences by characteristic.

Characteristic				Drive-through site: throat swab					Clinic: throat swab									
			Mean (SD)		Median (IQR)		*P* value^a^		Mean (SD)			Median (IQR)					*P* value	
Overall				3.8 (1.3)		4 (3-5)		N/A^b^		3.5 (1.4)			4 (3-5)					N/A
**Gender**								>.99								.57		
	Female			3.8 (1.3)		4 (3-5)				3.4 (1.4)			4 (2-5)					
	Male			3.9 (1.3)		4 (3-5)				3.6 (1.4)			4 (3-5)					
	Other			3.7 (1.4)		4 (3-5)				3.6 (1.2)			3.5 (3-5)					
**Age (years)**								.047								>.99		
	18-29			3.7 (1.4)		4 (3-5)				3.5 (1.4)			4 (3-5)					
	30-49			3.9 (1.3)		4 (3-5)				3.5 (1.4)			4 (3-5)					
	50-64			4 (1.3)		4 (3-5)				3.4 (1.4)			4 (2-5)					
	≥65			3.9 (1.2)		4 (3-5)				3.6 (1.3)			4 (3-5)					
**Race/ethnicity**								>.99								.99		
	Hispanic			3.9 (1.3)		4 (3-5)				3.6 (1.4)			4 (3-5)					
	Asian/Pacific Islander			3.7 (1.4)		4 (3-5)				3.6 (1.4)			4 (2.75-5)					
	Non-Hispanic Black			3.7 (1.4)		4 (3-5)				3.5 (1.5)			4 (2-5)					
	Non-Hispanic White			3.9 (1.3)		4 (3-5)				3.4 (1.4)			3 (2-5)					
	Other			3.6 (1.4)		4 (3-5)				3.4 (1.4)			3 (2.25-5)					
**Education**								.69								>.99		
	College, postgraduate, or professional school			3.8 (1.3)		4 (3-5)				3.4 (1.4)			4 (2-5)					
	Some college, associate's degree, or technical school			4 (1.2)		5 (3-5)				3.6 (1.4)			4 (3-5)					
	High school/GED^c^			3.6 (1.4)		4 (3-5)				3.5 (1.4)			4 (3-5)					
	Did not finish high school			3.8 (1.3)		4 (3-5)				3.5 (1.5)			4 (2.5-5)					
**Annual income (US $)**								>.99								.82		
	<24,000			3.8 (1.3)		4 (3-5)				3.7 (1.4)			4 (3-5)					
	24,000 to <50,000			3.9 (1.3)		4 (3-5)				3.5 (1.4)			4 (3-5)					
	50,000 to <75,000			3.9 (1.2)		4 (3-5)				3.5 (1.4)			4 (3-5)					
	≥75,000			3.9 (1.3)		5 (3-5)				3.4 (1.5)			3 (2-5)					
	Don't know			3.6 (1.3)		4 (3-5)				3.4 (1.3)			3 (3-5)					
**How likely do you think it is you have COVID-19 now?**								.001								.003		
	Very unlikely			3.6 (1.5)		4 (3-5)				3.3 (1.5)			3 (2-5)					
	Unlikely			3.8 (1.3)		4 (3-5)				3.5 (1.4)			4 (3-5)					
	Somewhat likely			4 (1.2)		4.5 (3-5)				3.7 (1.3)			4 (3-5)					
	Likely/very likely			4 (1.3)		5 (3-5)				3.7 (1.4)			4 (3-5)					
**Stigma index score**								>.99								.70		
	0			3.9 (1.2)		4 (3-5)				3.6 (1.3)			4 (3-5)					
	1-2			3.8 (1.4)		4 (3-5)				3.4 (1.4)			3 (2-5)					
	≥3			3.7 (1.4)		4 (3-5)				3.5 (1.5)			4 (2-5)					
**Knowledge index score**								>.99								>.99		
	<12			3.9 (1.3)		4 (3-5)				3.6 (1.4)			4 (3-5)					
	12-13			3.8 (1.3)		4 (3-5)				3.5 (1.4)			4 (2-5)					
	14			3.8 (1.3)		4 (3-5)				3.4 (1.4)			4 (2-5)					
**Symptoms**								.21								.65		
	1 or more symptoms			3.9 (1.3)		4 (3-5)				3.6 (1.4)			4 (3-5)					
	None			3.7 (1.4)		4 (3-5)				3.4 (1.4)			4 (2-5)					

^a^All *P* values were adjusted for multiple comparisons using the Bonferroni-Holm method.

^b^N/A: not applicable.

^c^GED: General Education Development.

**Table 4:** Stated willingness of internet-using adults aged ≥18 years in the United States in March 2020 to use home and clinic blood sample specimen collection testing modalities on themselves as part of a COVID-19 research study (N=1435). All survey questions were 5-point Likert scale items where 1=strongly disagree, 2=disagree, 3=undecided, 4=agree, 5=strongly agree. Kruskal-Wallis tests and Spearman rank correlation coefficients were used to assess response differences by characteristic.

Characteristic			Home: blood sample						Clinic: blood sample										
				Mean (SD)		Median (IQR)		*P* value^a^		Mean (SD)				Median (IQR)					*P* value
Overall			3.8 (1.3)		4 (3-5)		N/A^b^		3.6 (1.3)				4 (3-5)					N/A	
**Gender**							>0.99										>0.99		
	Female		3.8 (1.3)		4 (3-5)				3.5 (1.3)				4 (3-5)						
	Male		3.9 (1.3)		4 (3-5)				3.7 (1.3)				4 (3-5)						
	Other		4.1 (1.3)		5 (4-5)				3.6 (1.2)				4 (3-5)						
**Age (years)**							<0.001										>0.99		
	18-29		3.5 (1.4)		4 (2-5)				3.5 (1.4)				4 (3-5)						
	30-49		4.0 (1.2)		4 (3-5)				3.7 (1.3)				4 (3-5)						
	50-64		4.1 (1.1)		5 (3-5)				3.6 (1.3)				4 (3-5)						
	≥65		4.0 (1.2)		4 (3-5)				3.7 (1.3)				4 (3-5)						
**Race/ethnicity**							>0.99										>0.99		
	Hispanic		3.6 (1.4)		4 (3-5)				3.7 (1.2)				4 (3-5)						
	Asian/Pacific Islander		3.8 (1.3)		4 (3-5)				3.6 (1.3)				4 (3-5)						
	Non-Hispanic Black		3.6 (1.4)		4 (3-5)				3.5 (1.4)				4 (2-5)						
	Non-Hispanic White		3.9 (1.3)		4 (3-5)				3.5 (1.3)				4 (3-5)						
	Other		3.7 (1.3)		4 (3-5)				3.6 (1.3)				4 (3-5)						
**Education**							>0.99										>0.99		
	College, postgraduate, or professional school		4.0 (1.3)		4 (3-5)				3.6 (1.3)				4 (3-5)						
	Some college, associate's degree, or technical school		3.9 (1.2)		4 (3-5)				3.4 (1.2)				4 (3-4)						
	High school/GED^c^		3.7 (1.3)		4 (3-5)				3.5 (1.4)				4 (2-5)						
	Did not finish high school		3.9 (1.2)		4 (3-5)				3.7 (1.3)				4 (3-5)						
**Annual income (US $)**							0.01										0.65		
	<24,000		3.9 (1.3)		4 (3-5)				3.7 (1.2)				4 (3-5)						
	24,000 to <50,000		4.0 (1.2)		4 (3-5)				3.5 (1.3)				4 (3-5)						
	50,000 to <75,000		3.8 (1.3)		4 (3-5)				3.7 (1.3)				4 (3-5)						
	≥75,000		4.2 (1.1)		5 (4-5)				3.7 (1.3)				4 (3-5)						
	Don't know		3.5 (1.4)		4 (3-5)				3.5 (1.3)				3 (3-5)						
**How likely do you think it is you have COVID-19 now?**							>0.99										>0.99		
	Very unlikely		3.9 (1.3)		4 (3-5)				3.4 (1.4)				4 (2-5)						
	Unlikely		3.8 (1.3)		4 (3-5)				3.6 (1.3)				4 (3-5)						
	Somewhat likely		3.8 (1.3)		4 (3-5)				3.7 (1.2)				4 (3-5)						
	Likely/very likely		3.5 (1.5)		4 (3-5)				3.6 (1.5)				4 (3-5)						
**Stigma index score**							>0.99										>0.99		
	0		3.9 (1.3)		4 (3-5)				3.7 (1.3)				4 (3-5)						
	1-2		3.8 (1.4)		4 (3-5)				3.5 (1.4)				4 (2-5)						
	≥3		3.7 (1.4)		4 (2-5)				3.4 (1.4)				4 (2-5)						
**Knowledge index score**							>0.99										>0.99		
	<12		3.8 (1.3)		4 (3-5)				3.6 (1.3)				4 (3-5)						
	12-13		3.9 (1.3)		4 (3-5)				3.6 (1.3)				4 (3-5)						
	14		3.8 (1.3)		4 (3-5)				3.5 (1.4)				4 (3-5)						
**Symptoms**							>0.99										>0.99		
	1 or more symptoms		3.8 (1.3)		4 (3-5)				3.6 (1.3)				4 (3-5)						
	None		3.9 (1.3)		4 (3-5)				3.5 (1.3)				4 (3-5)						

^a^*P* values were adjusted for multiple comparisons using the Bonferroni-Holm method.

^b^N/A: not applicable.

^c^GED: General Education Development.

The mean willingness rating (Likert 5-point scale) ranged from 3.5 (clinic throat swab, median 4) to 4.5 (home saliva sample, median 5; Table 5). Compared to the participants’ willingness to use a home saliva sample, there was a medium effect size in willingness to use a home test blood sample (Cohen *d*=0.568; 95% CI 0.510-0.627) and willingness to use a drive-through throat swab (Cohen *d*=0.567; 95% CI 0.507-0.627). There was a large effect size in willingness to use a clinic for either a throat swab (Cohen *d*=0.802; 95% CI 0.732-0.872) or a blood sample (Cohen *d*=0.776; 95% CI 0.706-0.847) compared to using a home test saliva sample. A similar pattern was seen in comparisons between willingness to use different testing modalities for children.

**Table 5:** Stated willingness of internet-using adults aged ≥18 years in the United States in March 2020 to use specimen collection modalities on themselves and their children as part of a COVID-19 research study and relative effect sizes. All survey questions were 5-point Likert scale items where 1=strongly disagree, 2=disagree, 3=undecided, 4=agree, and 5=strongly agree. All *P*<.001.

Specimen collection modality		n (%)	Mean (SD)	Median (IQR)	Cohen *d*	95% CI
**Willing to use on oneself (N=1435)**						
	Home: saliva sample	1435 (100.0)	4.5 (0.9)	5 (4-5)	Reference	N/A^a^
	Home: throat swab	1435 (100.0)	4.4 (1.9)	5 (4-5)	0.114	0.085 to 0.144
	Home: blood sample	1434 (99.9)	3.8 (1.3)	4 (3-5)	0.568	0.510 to 0.627
	Drive-through site: throat swab	1435 (100.0)	3.8 (1.3)	4 (3-5)	0.567	0.507 to 0.627
	Clinic: throat swab	1435 (100.0)	3.5 (1.4)	4 (3-5)	0.802	0.732 to 0.872
	Clinic: blood sample	1434 (99.9)	3.6 (1.3)	4 (3-5)	0.776	0.706 to 0.847
**Willing to use on one’s children (n=385)**						
	Home: saliva sample	385 (100.0)	4.1 (1.3)	5 (4-5)	Reference	N/A
	Home: throat swab	385 (100.0)	4.0 (1.3)	5 (3-5)	0.113	–0.029 to 0.254
	Home: blood sample	334 (87.8)	3.4 (1.5)	3 (2-5)	0.454	0.388 to 0.520
	Drive-through site: throat swab	385 (100.0)	3.4 (1.5)	4 (2-5)	0.517	0.373 to 0.660
	Clinic: throat swab	385 (100.0)	3.1 (1.5)	3 (2-5)	0.742	0.596 to 0.889
	Clinic: blood sample	334 (87.8)	3.0 (1.5)	3 (2-4)	0.851	0.768 to 0.933

^a^N/A: not applicable.

## Discussion

### Principal Findings

Response to the SARS-CoV-2 epidemic in the United States has been hampered by insufficient testing both for diagnosing persons and for public health assessments to describe the epidemiology of infection. Critical shortages of reagents, other supply chain issues, and lack of availability of health care workers have led to gaps in testing, and it is critical to diversify testing methods. Options include alternative specimens to be tested and specimen collection locations. Further, it is important to understand which testing options are best suited to which purposes (eg, clinical care, population research, and screening versus diagnosis). Self-collection of specimens at home has proven to be an acceptable approach in other infectious disease testing, and it could play an important role in the response to the SARS-CoV-2 epidemic in the United States.

Results from this study indicate that a large majority of adults would be willing to participate in a research study about SARS-CoV-2 infection or immune experience by collecting specimens at home and mailing them to a laboratory for testing. There was some preference for certain specimen collection modalities (saliva samples and throat swabs were preferred over blood samples); however, these differences were largely driven by preference for remote home specimen collection methods versus methods that would require visits to clinical or laboratory locations for testing. Testing location and specimen collection preferences were consistent across demographic groups and other characteristics of interest. These results are similar to literature reports that indicate that home specimen collection options are preferred to clinic-based testing methods for HIV and STI screening [11,15,16].

These findings are important for the design of forthcoming epidemiologic studies of SARS-CoV-2 infection. Cross-sectional and cohort follow-up studies will depend on testing biological specimens to accurately measure disease prevalence, incidence, and recovery among participants. Our results indicate that study designs that use home specimen collection experience increased participation and higher retention compared to study designs that involve traveling to a drive-through site or clinic. The high willingness to use home specimen collection methods across demographic groups and other subgroups suggests that studies incorporating home specimen collection may be less susceptible to participation bias than designs requiring the collection of biological specimens in clinical settings.

The use of home specimen collection methods can also help ensure that research activities do not have a negative effect on adherence to current health guidance. Home specimen collection can be incorporated within the context text of social distancing guidelines [17], which can enable study participants to maximize their individual contributions to slowing the spread of COVID-19 disease. Similarly, reducing the contact between study participants and health care workers improves health care worker safety by reducing their risk of exposure. Further, such options can reduce the overall burden on health care providers and clinics that may not have the capacity to collect specimens as part of ongoing research. For instance, the current shortage of personal protective equipment among health care workers in the United States has been well documented [18], and the incorporation of home specimen collection can ensure that these resources are preserved for use in clinical care.

Willingness of people to use home specimen collection kits for research offers promising opportunities to conduct representative and timely research about SARS-CoV-2 infection and immune response. However, it is important to recognize that the collection of home specimens will also require rigorous testing to ensure that the laboratory results obtained provide accurate indications of SARS-CoV-2 infection and immune response, the kits are safe for participants to use, and the specimens are sufficiently robust to maintain validity after the process of return shipping to laboratories for analysis. It is important to distinguish between home collection of specimens that are mailed back to laboratories for analysis from the separate field of home testing, in which participants collect their own specimen, apply it to a test device, and interpret the results at home.

One reason that the use of home specimen collection may be associated with high willingness to participate in research related to SARS-CoV-2 infection is that the virus is highly infectious, and there is meaningful risk associated with entering clinical settings where people with symptoms of COVID-19 are congregated. Unlike testing as part of research studies for less contagious infectious diseases or for other types of disease, potential research participants who do not have symptoms may be particularly reluctant to report to clinical locations for screening. Relatedly, if home collection of specimens is developed and validated, it will be possible to conduct epidemiological studies that can both reach people in diverse geographic areas, including rural areas, and allow research to be conducted without exposing participants to potential harms associated with going to research sites that may result in their exposure to SARS-CoV-2.

### Limitations

Our study results have several limitations. First, we assessed willingness using Likert scale items, which limits the ability to determine the magnitude of preference for one test modality over the other for any individual. However, Likert data are especially well suited for assessing the direction of preference, which is a relevant outcome given our desire to understand the potential impact of offering home specimen collection in research settings. Second, our recruitment methods targeted social media users, and our convenience sample may not be representative of all US adults. Third, opinions regarding testing for SARS-CoV-2 may change over time, given the rapidly shifting nature of public perception regarding the epidemic, and updates are merited to ensure that participant preferences remain stable. Finally, we know that there have historically been disconnects between expressed willingness to use self-testing or at-home specimen collection options for infectious diseases and the actual uptake of these highly acceptable devices [19]. However, compared to historical examples of the introduction of at-home tests before the advent of telemedicine, testing for SARS-CoV-2 infection with specimens collected at home for research or clinical purposes may be more acceptable given the broad availability of telehealth clinical services. These services may be used to provide support for participants who have questions about collecting specimens at home or to observe the self-collection of specimens at home until data are developed to document the sufficiency and quality of specimens collected at home.

### Conclusions

Large scale population-based research and testing is needed to provide the epidemiologic data necessary to guide our public health response to the COVID-19 pandemic. Home specimen collection strategies should be considered to achieve the highest levels of participant engagement and retention, reduce the burden of specimen collection in overloaded health care settings, and reduce potential exposure of research participants to SARS-CoV-2 in research settings.

## Abbreviations

STI: sexually transmitted infection
